# Hippocampal T2 hyperintensities on 7 Tesla MRI^☆^

**DOI:** 10.1016/j.nicl.2013.08.003

**Published:** 2013-08-17

**Authors:** Susanne J. van Veluw, Laura E.M. Wisse, Hugo J. Kuijf, Wim G.M. Spliet, Jeroen Hendrikse, Peter R. Luijten, Mirjam I. Geerlings, Geert Jan Biessels

**Affiliations:** a Department of Neurology, Brain Center Rudolf Magnus, University Medical Center Utrecht, Utrecht, The Netherlands; b Julius Center for Health Sciences and Primary Care, University Medical Center Utrecht, Utrecht, The Netherlands; c Image Sciences Institute, University Medical Center Utrecht, Utrecht, The Netherlands; d Department of Pathology, University Medical Center Utrecht, Utrecht, The Netherlands; e Department of Radiology, University Medical Center Utrecht, Utrecht, The Netherlands

**Keywords:** Hippocampal sulcal cavities, Neuropathology, Microinfarcts, Fluid-attenuated inversion recovery, Perivascular spaces

## Abstract

Hippocampal focal T2 hyperintensities (HT2Hs), also referred to as hippocampal sulcal cavities, are a common finding on Magnetic Resonance (MR) images. There is uncertainty about their etiology and clinical significance. In this study we aimed to describe these HT2Hs in more detail using high resolution 7 Tesla MR imaging, addressing 1) the MR signal characteristics of HT2Hs, 2) their occurrence frequency, 3) their location within the hippocampus, and 4) their relation with age. We also performed an explorative post-mortem study to examine the histology of HT2Hs.

Fifty-eight persons without a history of invalidating neurological or psychiatric disease (mean age 64 ± 8 years; range 43–78 years), recruited through their general practitioners, were included in this study. They all underwent 7 Tesla MRI, including a T1, T2, and FLAIR image. MR signal characteristics of the HT2Hs were assessed on these images by two raters. Also, the location and number of the HT2Hs were assessed. In addition, four formalin-fixed brain slices from two subjects were scanned overnight. HT2Hs identified in these slices were subjected to histopathological analysis.

HT2Hs were present in 97% of the subjects (median number per person 10; range 0–20). All HT2Hs detected on the T2 sequence were hypointense on T1 weighted images. Of all HT2Hs, 94% was hypointense and 6% hyperintense on FLAIR. FLAIR hypointense HT2Hs were all located in the vestigial sulcus of the hippocampus, FLAIR hyperintense HT2Hs in the hippocampal sulcus or the gray matter. Post-mortem MRI and histopathological analysis suggested that the hypointense HT2Hs on FLAIR were cavities filled with cerebrospinal fluid. A hyperintense HT2H on FLAIR proved to be a microinfarct upon microscopy.

In conclusion, hippocampal T2Hs are extremely common and unrelated to age. They can be divided into two types (hypo- and hyperintense on FLAIR), probably with different etiology.

## Highlights

• With 7T MRI we found many hippocampal T2 hyperintensities in a healthy population.
• Two subtypes can be distinguished: hippocampal sulcal cavities and microinfarcts.
• They appear to be extremely common and unrelated to age.

## 1 Introduction

On MR images of the hippocampus, focal T2 hyperintensities, also referred to as hippocampal sulcal cavities, are a common finding. They are reported to be hypointense on T1 and hyperintense on T2 weighted images. Most studies hypothesized that these T2 hyperintensities are anatomic variations, thought to occur during development as a result of incomplete folding of the hippocampus, leading to small fluid collections – i.e. cavities – in the vestigial sulcus of the hippocampus [1], [2]. Others, however, argue that the T2 hyperintensities are lesions related to ischemic or hypoxic events [3], [4]. This ambiguity concerning their origin is also reflected in the various names they received including hippocampal sulcal cavities, low signal foci, and hyperintense lesions. In our view it is best to name them according to their MR features, without prior assumptions on their etiology. In the present paper we will therefore refer to them as focal hippocampal T2 hyperintensities, abbreviated as HT2Hs.

A recent review of the MR literature reported that HT2Hs are very common with a mean prevalence of 47% in healthy older controls, though reported numbers vary considerably [5]. Also, the relation of HT2Hs with age [1], [6], [7], vascular risk factors [5], or memory [4], [2], [8] is not clear. Until now, HT2Hs have mostly been investigated using T1 or T2 weighted images that cannot differentiate between fluid-filled hippocampal cavities and gliotic ischemic lesions. Furthermore, they have solely been described on conventional field strength MRI, often using thick slices (5 mm or more), which might have led to an underestimation of their occurrence.

In this study we describe HT2Hs at 7 Tesla MRI using T1, T2, and fluid-attenuated inversion recovery (FLAIR) images in people without known neurological disease. We aimed to describe 1) the MR signal characteristics of HT2Hs on T1 and FLAIR, 2) their occurrence, 3) their location within the hippocampus, and 4) their relation with age, blood pressure, cognition, and other brain MRI markers. Finally we performed an explorative post-mortem study in brain material from other subjects to examine the histology of these HT2Hs.

## 2 Material and methods

### 2.1 Study population

**Fig. 1:**
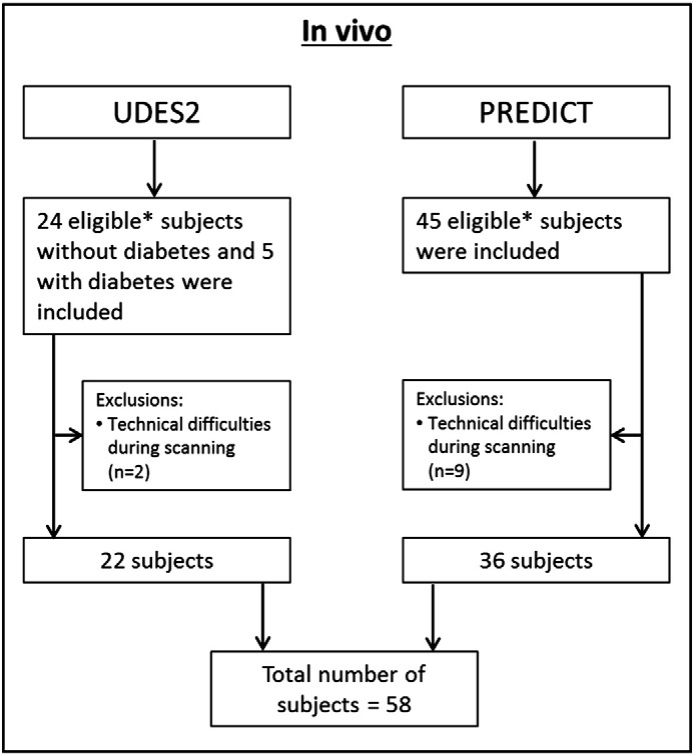
Flowchart of study participants. *Subjects were eligible for the current study when they met the following criteria: 1) age above 40, 2) no known psychiatric or neurological disorder that could affect cognitive functioning, 3) a Mini-Mental State Examination (MMSE) score of 25 or higher, 4) no contraindications for 7 Tesla MRI (e.g. metal in or on their bodies, claustrophobia, and 5) T1, T2, and FLAIR.

Subjects were recruited through their general practitioners as part of two related cohorts with similar experimental work-up (PREDICT-MR; a prospective cohort MR study of individuals attending their general practitioner, not selected for presence of specific medical conditions [9] and UDES2; a study on MRI correlates of type 2 diabetes mellitus that included both participants with and without diabetes [10]). From these studies we selected all participants that met the following criteria: 1) age above 40, 2) no known psychiatric or neurological disorder that could affect cognitive functioning, 3) a Mini-Mental State Examination (MMSE) score of 25 or higher, 4) no contraindications for 7 Tesla MRI (e.g. metal in or on their bodies, claustrophobia), and 5) 7 Tesla T1, T2, and FLAIR. From the UDES2, all controls and a random sample of the patients with diabetes, in accordance with the prevalence of type 2 diabetes mellitus in the Dutch population (14%), were eligible for the present study. The total study sample comprised 58 subjects (Fig. 1).

PREDICT-MR and UDES2 were approved by the medical ethics committee of the University Medical Center Utrecht (UMCU), and all subjects gave written informed consent.

All participants underwent a standardized evaluation, including medical history, physical (e.g. blood pressure), neurological, and neuropsychological examination. On the same day all participants underwent conventional (PREDICT-MR 1.5 T; UDES2 3 T) and 7 Tesla MRI. With respect to the neuropsychological examination, in this study we primarily focused on memory. A composite z-score for memory was calculated based on the immediate, delayed recall and the retention score of the 15-word learning test (a modification of the Rey Auditory Verbal Learning test). Composite z-scores were computed by converting raw scores to standardized z-scores and averaging them across all subtests.

### 2.2 MRI scanning protocol

Scans were acquired on a whole-body 7 Tesla MR system (Philips Healthcare, Cleveland, OH, USA) with a volume transmit and 16-channel receive head coil (Nova Medical, Wilmington, MA, USA). Subjects included in the study later than May 2011 were scanned with a volume transmit and 32-channel receive head coil (Nova Medical). The standardized protocol included the following sequences:

– Volumetric (3D) magnetization prepared (MP-) FLAIR with an acquired, isotropic resolution of 0.8 × 0.8 × 0.8 mm^3^, repetition time (TR) = 8000 ms, nominal echo time (TE) = 300 ms using constant low refocusing angles of 70°, inversion time (TI) = 2325 ms, matrix size = 312 × 304. Scan duration 12 min 48 s.
– 3D T2 weighted turbo-spin echo (TSE) with an acquired, isotropic resolution of 0.7 × 0.7 × 0.7 mm^3^, TR = 3158 ms, nominal TE = 346 ms with a variable refocusing flip angle sweep, leading to an equivalent TE (for T2 contrast) of approximately 57 ms for gray and white matter, matrix size = 356 × 357. Scan duration 10 min 15 s.
– 3D T1 weighted sequence with an isotropic resolution of 1.0 × 1.0 × 1.0 mm^3^, flip angle 8°, TR = 4.8 ms, TE = 2.2 ms, TI = 1240 ms, TR of the inversion pulses = 3500 ms, matrix size = 200 × 250. Scan duration 1 min 57 s.

### 2.3 Visual rating and volume assessment

Two raters (SvV and LW), blinded to subject information, independently assessed MR signal characteristics, number, and location of the HT2Hs on T2, T1, and FLAIR 7 Tesla images. In case of disagreement a consensus meeting was held and if necessary discussed with a neuroradiologist (JH).

One rater (LW) assessed total hippocampal volume and volume of HT2Hs on consecutive slices of the T2 weighted 7 Tesla images using an in-house developed software program (by HK), based on MeVisLab (MeVis Medical Solutions AG, Bremen, Germany [11]) [9]. The volume of HT2Hs was assessed again after a four-week interval in a randomly chosen sample of 15 subjects to establish intra-rater reliability. The intraclass correlation coefficient for total hippocampal volume was 0.98 [9].

White matter hyperintensities (WMHs) and lacunar infarcts were assessed on 3 Tesla 2D FLAIR (voxel size 1.0 × 1.3 × 3.0 mm^3^) and 3D T1 (voxel size 1.0 × 1.0 × 1.0 mm^3^) (UDES2) or 1.5 Tesla 3D FLAIR and 3D T1 (PREDICT-MR; both with voxel size 1.1 × 1.1 × 1.1 mm^3^) using the age-related white matter changes (ARWMC) scale [12]. We choose not to use the 7 Tesla scans for these ratings, because the ARWMC scale has not yet been applied to scans of this field strength. Two independent raters rated WMHs and lacunar infarcts. In case of disagreement a consensus meeting was held.

### 2.4 Post-mortem study

Post-mortem brain tissue of two patients, who were autopsied in the UMCU, was scanned in an overnight scanning session as part of another study [13]. They were included as an explorative post-mortem study to examine the nature of the observed in vivo HT2Hs with histology. Details of the ex vivo MRI, sampling, and histopathology procedure are described elsewhere [13]. In short, four formalin-fixed 10-mm thick coronal brain slices were placed in a plastic container filled with 4% formaldehyde (formalin) and scanned overnight at 7 T. The standard post-mortem protocol consisted of a T2 weighted and FLAIR sequence, both with voxel sizes of 0.4 × 0.4 × 0.4 mm^3^. HT2Hs were scored by one rater, sampled, and subjected to histopathological analysis. Blocks of tissue were processed and embedded in paraffin, after which 6-μm thick serial sections were cut using a microtome. Standard histologic staining (HE and LFB) was performed on these sections. A neuropathologist (WS) examined the sections under the microscope. The use of the post-mortem material for this study was in accordance with local regulations and approved by the local medical ethics committee.

### 2.5 Statistical analyses

The intraclass correlation coefficient (ICC) was used to calculate the inter-rater reliability in rating number of HT2Hs. The ICC and Dice similarity coefficient (DSC) were used to assess the intra-rater reliability for assessing the volume of the HT2Hs. Linear or logistic regression analyses (adjusted for gender) was performed to analyze the relation between HT2Hs and age.

We also performed exploratory linear or logistic regression analyses (adjusted for age and gender) to investigate the relation with mean arterial pressure (MAP), cognitive performance, hippocampal volume, and WMHs and lacunar infarcts.

All data analyses were performed using SPSS version 20.0 (SPSS, Inc, Chicago, IL).

## 3 Results

### 3.1 In vivo

**Fig. 2:**
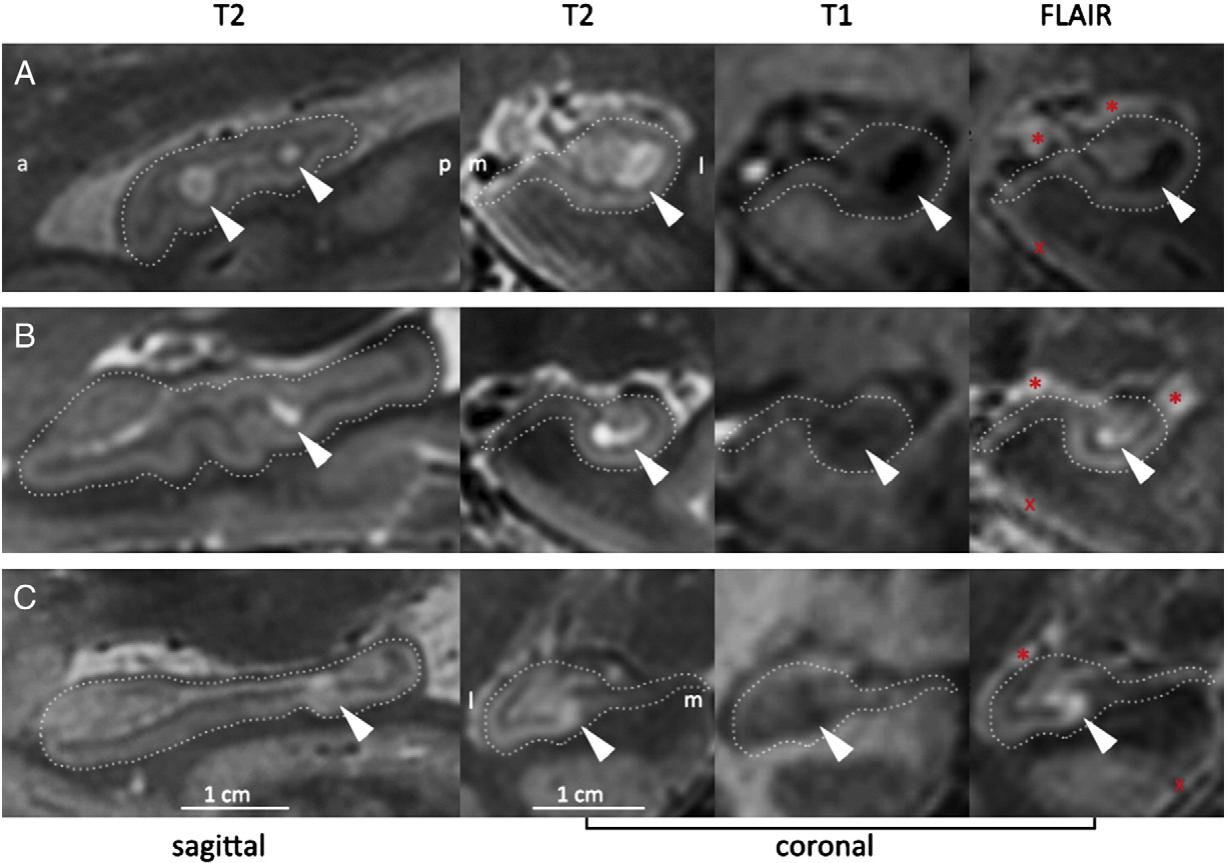
Hippocampal T2 hyperintensities on 7 Tesla MRI. Examples of hippocampal T2 hyperintensities that were hypointense (A) or hyperintense (BC) on 7 Tesla FLAIR. The left figure on each row is sagittal, the other three are coronal. For clarity, the hippocampus is outlined. Most HT2Hs appeared round or ovoid, but the ones that were hyperintense on FLAIR sometimes had a different shape. * indicates the choroid plexus, which is also hyperintense on FLAIR, but situated outside the hippocampus. Therefore it does not interfere with a reliable rating of focal HT2Hs within the hippocampus. X indicates the fluid — parenchyma border, which is also hyperintense on FLAIR. This hyperintense rim, characteristic for 7 Tesla FLAIR, is located along the outer surface of the brain and therefore does not interfere with a reliable rating of focal HT2Hs within the hippocampus. a: anterior; p: posterior; m: medial; l: lateral. The whole-brain 7 Tesla FLAIR scan of the participant whose hippocampus is depicted in panel C is available as a movie in the online supplementary material. Supplementary material Whole-brain FLAIR of a healthy 54-year old individual. This particular individual has a focal hippocampal FLAIR hyperintensity, possibly due to ischemia (best visible on sagittal view at 0:29min, and on coronal view at 0:34min). More details on 7T FLAIR contrast provided at [14]. This movie can be found at: http://www.youtube.com/watch?v=EaF_wbYlYc0.

Subject characteristics are displayed in Table 1. The study sample consisted of 58 subjects with a mean age of 64 ± 8 years, 64% females and a mean MMSE of 28.8 ± 1.2. HT2Hs were found to be hypointense on T1 and either hypo- or hyperintense on FLAIR (Fig. 2).

**Table 1:** Subject characteristics.

	Subjects (n = 58)
Age in years	64 ± 8
Gender (% male)	21 (36)
MMSE	29 [25–30]
MAP	101 ± 9
Diabetes (%)	6 (10)

Mean ± SD, n(%), or median [range] are shown.

MMSE = Mini-Mental State Examination.

MAP = mean arterial pressure: diastolic pressure + 1/3 (systolic–diastolic pressure).

The inter-rater ICC for all HT2Hs was 0.79 (95% CI 0.57; 0.89). The inter-rater ICC for FLAIR hypointensities was 0.73 (95% CI 0.56; 0.84) and for FLAIR hyperintensities 0.27 (95% CI 0.02; 0.49). The intra-rater ICC for HT2H volume assessment was 0.98 (95% CI 0.94; 0.99) and the DSC was 0.75 (95% CI 0.69; 0.80). Other structures that are hyperintense on 7 Tesla FLAIR, in particular the choroid plexus and the fluid — parenchyma border [14], could be readily distinguished from HT2Hs, based on shape and location (Fig. 2).

HT2Hs were present in almost all subjects with a median number per person of 10 (Table 2). Of the total number of HT2Hs, 94% was hypointense on FLAIR, and 6% was hyperintense on FLAIR. Most HT2Hs appeared round or ovoid on MR, but the ones that were hyperintense on FLAIR sometimes had a different shape, see for example Fig. 2.

**Table 2:** Characteristics and prevalence of hippocampal T2 hyperintensities.

		Any HT2Hs	HT2Hs, hypointense on FLAIR	HT2Hs, hyperintense on FLAIR
Per person	HT2Hs present	97%	97%	36%
	Median number (range)	10 (0–20)	9.5 (0–19)	0 (0–5)
	Mean total volume/person in μL	40.8 ± 4.75	38.5 ± 3.62	2.3 ± 0.54
Per HT2H	N; % of total number of HT2Hs	571; 100%	535; 94%	36; 6%
	Diameter (range) in mm	1–10	1–10	1–4
	Mean volume/single HT2H in μL	3.9 ± 2.49	3.9 ± 2.75	3.2 ± 2.08

**Fig. 3:**
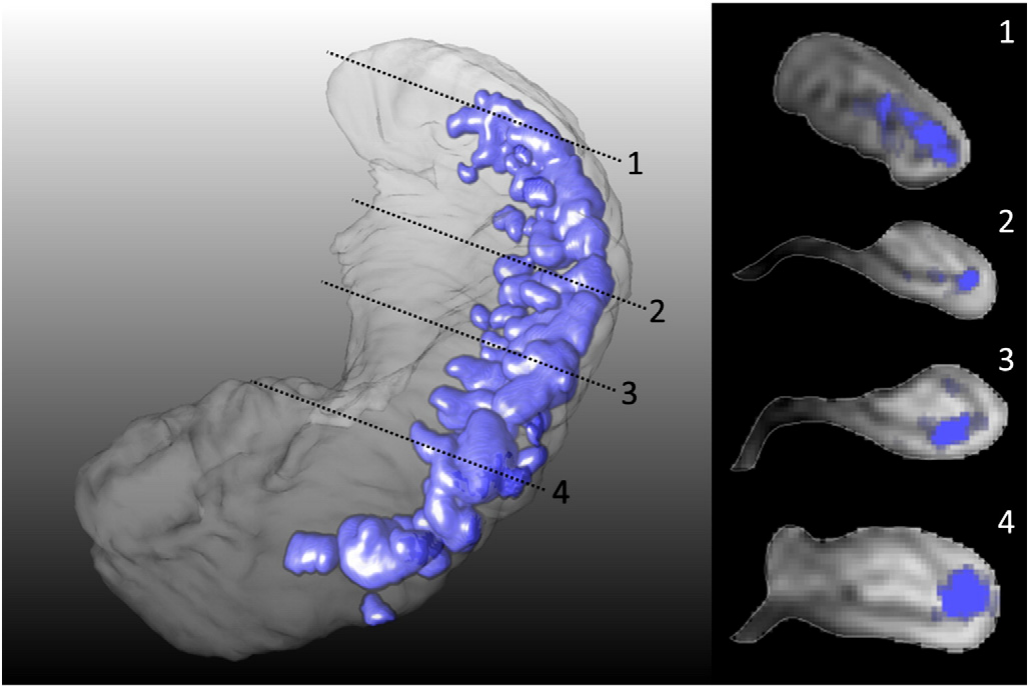
3D representation of hippocampal T2 hyperintensities. Left: probabilistic segmentations of FLAIR hypointense HT2Hs of a representational subset were registered to the hippocampus of one subject. The 3D distribution of HT2Hs is shown in purple, all FLAIR hypointense HT2Hs were located in the vestigial sulcus in the full length of the hippocampus, and a vast majority in the ventrolateral flexion points of CA1. Right: cross-sections of the hippocampus shown on the left.

The HT2Hs that appeared hypointense on FLAIR were all located in the vestigial sulcus of the hippocampus along the full longitudinal axis, and with high frequency at the ventrolateral flexion point of the cornu ammonis (Fig. 3). Of the HT2Hs that appeared hyperintense on FLAIR, 25% were located in the gray matter, and 75% appeared to be located in the vestigial sulcus.

**Fig. 4:**
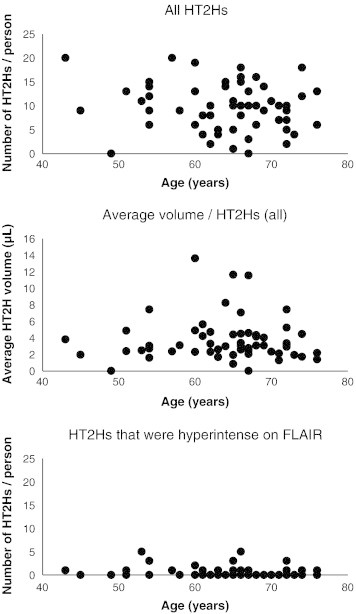
Scatterplots of number or volume of hippocampal T2 hyperintensities against age.

Increasing age was not associated with a higher number or average volume of HT2Hs (β = − 0.12 95% CI − 0.38; 0.15 and β = − 0.03 95% CI − 0.31; 0.25, respectively) (Fig. 4). An additional analysis between age and presence or volume of HT2Hs that appeared hyperintense on FLAIR also revealed no association (OR = 1.01 95% CI 0.94; 1.08 and β = − 0.10 95% CI − 0.35; 0.17, respectively). Also, no relation was found between age and mean HT2H volume (β = − 0.11 95% CI − 0.39; 0.17).

Higher MAP was associated with a higher number of all HT2Hs (β = 0.32 95% CI 0.05; 0.58), but not with presence of FLAIR hyperintense HT2Hs (OR = 0.98 95% CI 0.91; 1.05). Total HT2H number, or presence of FLAIR hyperintense HT2Hs, were not related to memory (β = − 0.04 95% CI − 0.29; 0.20; β = − 0.03 95% CI − 0.50; 0.44, respectively). Total hippocampal volume (β = 0.24 95% CI − 0.05; 0.53; OR = 0.39 95% CI 0.09; 1.70), WMHs (β = 0.08 95% CI − 0.19; 0.35; OR = 1.02 95% CI 0.99; 1.06), or presence of lacunar infarcts (β = 0.24 95% CI − 0.04; 0.51; OR = 1.60 95% CI 0.42; 6.18) were not associated with total HT2H number, or presence of FLAIR hyperintense HT2Hs.

### 3.2 Post-mortem study

**Fig. 5:**
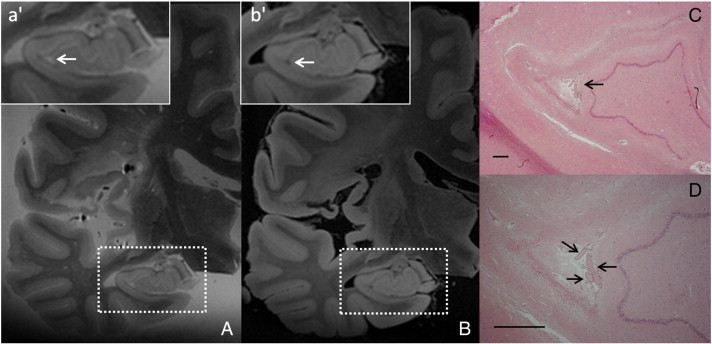
Hippocampal sulcal cavity. An HT2H, located in the vestigial sulcus, was identified on the ex vivo T2 weighted MR image (voxel size 0.4 × 0.4 × 0.4 mm^3^) of a formalin-fixed brain slice (A; box enlarged in a'). The corresponding location was hypointense on FLAIR (voxel size 0.4 × 0.4 × 0.4 mm^3^) (B; box enlarged in b'). After histopathological analysis this MR finding proved to be a cavity filled with fluid (C; arrow). Note the presence of the blood vessels in the fluid-filled cavity (D) (CD; HE stain; scale bars indicate 1 mm).

**Fig. 6:**
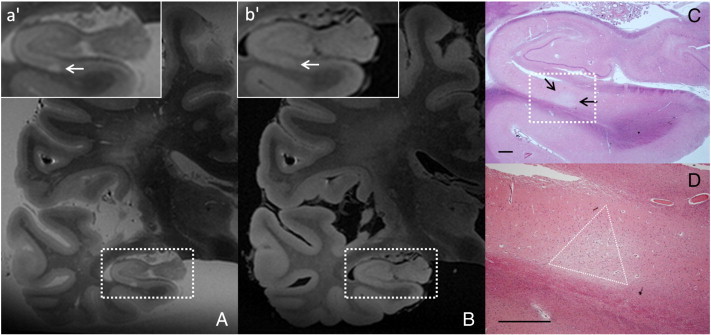
Hippocampal microinfarct. An HT2H, located in the gray matter, was identified on the ex vivo T2 weighted MR image (voxel size 0.4 × 0.4 × 0.4 mm^3^) of a formalin-fixed brain slice (A; box enlarged in a'). The corresponding location was also hyperintense on FLAIR, presuming this lesion to be ischemic (voxel size 0.4 × 0.4 × 0.4 mm^3^) (B; box enlarged in b'). After histopathological analysis this MR finding proved to be a microinfarct (C; arrows). The ischemic nature of this lesion becomes more clear when zoomed-in (D; triangle indicates microinfarct boundaries) (CD; HE stain; scale bars indicate 1 mm).

HT2Hs that were hypointense on FLAIR were found on all ex vivo MR images. One of these HT2Hs that appeared hypointense on FLAIR, was subjected to histopathological analysis. After examination of the corresponding section, this MR finding proved to be a cavity filled with cerebrospinal fluid (Fig. 5). Additionally, an HT2H in the gray matter that was hyperintense on FLAIR was found on the ex vivo MR images of one of the brain slices and was subjected to histopathological analysis as well. After examination of the corresponding section, this MR finding proved to be a microinfarct (Fig. 6). When zoomed-in a small delineated area of tissue pallor with loss of neurons and microglia infiltration could be discriminated, typical for a recent gliotic microinfarct.

## 4 Discussion

We investigated hippocampal T2Hs in a population without known neurological disease. The main findings of this study were that HT2Hs are extremely common, that their occurrence is unrelated to age, and that there appear to be two subtypes, probably with a different etiology.

In our study, HT2Hs were present in almost all subjects. A similar high frequency has been reported in another study [2]. However, the weighted mean of all previous studies (47%), as reported in a recent review [5], is much lower than our reported occurrence. The number of HT2Hs per person that we found also markedly exceeded previous reports [6], [2]. This indicates that the high resolution 7 Tesla MR images improve the sensitivity for detection.

We found that the HT2Hs that were hypointense on FLAIR appeared in the vestigial sulcus of the hippocampus and that they were not related to age. Both observations fit with the theory that they result from incomplete folding of the hippocampus and are normal phenomenon occurring during development. This was further supported in our explorative post-mortem study; the HT2H that was hypointense on FLAIR indeed appeared to be a cavity filled with cerebrospinal fluid, as already mentioned previously [1]. We found neither a relation between age and total volume load per person, nor a relation between age and mean volume per HT2H. Although it is possible that HT2Hs remain the same size during life, some HT2Hs were very large and an increase with age as a result of atrophy in surrounding tissue seems likely. However, the current study was not designed to investigate this latter hypothesis.

For some HT2Hs it proved to be difficult to distinguish between FLAIR hypo- and hyperintensity, leading to a lower rating reliability than for HT2H detection as such. Nevertheless, we did obtain evidence that these two different HT2Hs may have different meaning and etiology. Notably, the preferential location of the two HT2H types within the hippocampus differed. Our preliminary ex vivo data suggests that the FLAIR hyperintense HT2Hs that are located in the gray matter can be due to ischemia. The FLAIR hyperintense HT2Hs that were located in the vestigial sulcus should be further investigated with ex vivo MRI and histology to determine their true nature.

We did find a relation between blood pressure and number of HT2Hs. The meaning of this association is not yet clear. If HT2Hs would indeed develop under the influence of elevated blood pressure, the number of HT2Hs would also be expected to increase with age, as was not the case. Moreover, there was no relation between HT2H number and other markers of small vessel disease, and two earlier studies reported no significant relation between HT2Hs and hypertension [6], [8]. Future studies, including larger numbers of individuals of older age and/or with more advanced manifestations of small vessel disease, should investigate this in more detail. Such studies should also consider the distinction between FLAIR hypo- and hyperintense HT2Hs.

In conclusion, our 7 Tesla MR data showed that HT2Hs were even more common than previously reported. Even though we found more HT2Hs, no relation with age could be established. Future studies should look into the possibility that HT2Hs increase in size with age. Additionally, we found indications for an ischemic subtype, and more research is needed to investigate the clinical relevance of these ischemic HT2Hs, and their relation with vascular risk factors.
